# Is alcohol consumption in older adults associated with poor self-rated health? Cross-sectional and longitudinal analyses from the English Longitudinal Study of Ageing

**DOI:** 10.1186/s12889-015-1993-x

**Published:** 2015-07-24

**Authors:** Martin Frisher, Marina Mendonça, Nicola Shelton, Hynek Pikhart, Cesar de Oliveira, Clare Holdsworth

**Affiliations:** School of Pharmacy, Keele University, Keele, Staffordshire ST5 5BG UK; School of Physical and Geographical Sciences, Keele University, Keele, Staffordshire ST5 5BG UK; Department of Epidemiology and Public Health, University College, London, UK

**Keywords:** Alcohol, Epidemiology, Public Health, Longitudinal

## Abstract

**Background:**

Increases in alcohol related mortality and morbidity have been reported among older people in England over the last decade. There is, however, evidence that drinking is protective for some health conditions. The validity of this evidence has been questioned due to residual confounding and selection bias. The aim of this study is to clarify which drinking profiles and other demographic characteristics are associated with poor self-rated health among a community-based sample of older adults in England. The study also examines whether drinking designated as being “increasing-risk” or “higher-risk” is associated with poorer self-rated health.

**Method:**

This study used data from Wave 0, Wave 1 and Wave 5 of the English Longitudinal Study of Ageing [ELSA]. Logistic regression analysis, was used to examine the association between drinking profiles (based on quantity and frequency of drinking) and self-rated health, adjusting for gender, age, wealth, social class, education, household composition, smoking and body mass index [BMI].

**Results:**

Twenty percent of the sample reported drinking above the recommended level at wave 0. Rates of poor self-rated health were highest among those who had stopped drinking, followed by those who never drank. The rates of poor self-rated health among non-drinkers were significantly higher than the rates of poor self-rated health for any of the groups who reported alcohol consumption. In the adjusted logistic regression models there were no drinking profiles associated with significantly higher rates of poor self-rated health relative to occasional drinkers.

**Conclusions:**

Among those who drank alcohol, there was no evidence that any pattern of current alcohol consumption was associated with poor self-rated health, even after adjustment for a wide range of variables. The results associated with the stopped drinking profile indicate improvement in self-rated health can be associated with changes in drinking behaviour. Although several limitations of the study are noted, policy makers may wish to consider how these findings should be translated into drinking guidelines for older adults.

## Background

Age is an important determinant of drinking behaviours. Population data on alcohol consumption, such as the UK General Lifestyle Survey, show that older adults might consume less than younger groups, but are more likely to drink regularly [1]. Alcohol-related mortality is also highest at older age groups and increasing among the elderly, while stabilising at younger ages. For example, UK Office of National Statistics (ONS) data show that in 2012 the highest age-specific alcohol related mortality rates were for the age group 55–74, with rates of 40.1 per 100,000 and 19.8 per 100,000 for men and women respectively [2]. As a consequence of such findings alcohol consumption among the elderly has been identified as a growing public health issue [3].

While alcohol-related harms have increased in recent years, this has to be set against the evidence that drinking may be protective for some health conditions [4]. The possible health benefits accentuated at older ages include lowering the risk of dementia [5] and offering some protection against cardiovascular disease [6, 7] and asthma [8]. Evidence for the protective effects of moderate drinking on mortality is mixed, with continuing debate on the influence of confounding factors on the relationship [9, 10]. Older people also acknowledge the benefits of sociability associated with drinking [11]. The public health approach in the UK promotes responsible drinking, which seeks to balance the potential benefits of drinking against possible harms [12]. While drinking guidelines in the UK are not age-specific, clinical recommendations suggest a lower level of consumption for older people in response to recent data on harm among older people, and evidence on the effect of alcohol on body in later life [3]. Consumption of 14 or more drinks per week is associated with an increased risk of subsequent falls in adults aged 65 and over [13], and consumption of 18 or more drinks per week (when combined with a high level of education) is associated with recurrent falling (≥2 falls in a 6-month period) in the same age group [14].

Unravelling the causality between alcohol consumption and health is not straight-forward. In particular the U-or J-shape association between drinking and health is associated with the ‘sick-quitter’ hypothesis that people stop or moderate drinking because of ill-health, as well as known health risks associated with excessive consumption [15]. The causal association between drinking in later life and health could also reflect alcohol consumption as a marker of good health, rather than drinking determining health outcomes [16]. A further complication is that the association between alcohol and health could be influenced by other factors, in particular other life style behaviours that are determinants of health [17, 18] and socio-demographic characteristics [19]. A number of reasons why, for example, higher alcohol consumption might be associated with better health have been suggested [20]. More affluent people might access health services more frequently thereby reducing the impact of alcohol illness [21]. The impact of alcohol might also be mediated by diet and exercise although empirical evidence on this issue is mixed [22].

The aim of this paper is to examine a wide range of alcohol consumption patterns in relation to concurrent and future self-rated health. A secondary aim of the paper is to evaluate public health guidance for people who drink alcohol. Some of the analysis are therefore restricted to people who reported alcohol consumption in the last year and we used the people with the lowest level of alcohol consumption as the reference category. The study examines whether drinking designated by the UK government [23] as being “increasing-risk” - between 22 and 50 units per week [adult men] or between 15 and 35 units per week [adult women] - or “high-risk” - over 50 alcohol units per week [adult men] or over 35 units per week [adult women] - is associated with poorer self-rated health, in the context of other socio-economic and health indicators that have previously been found to be related to alcohol consumption and health. This identification of “risk” refers to the dangers to health that are potentially associated with drinking at these levels.

## Methods

### Data source and participants

**Fig. 1:**
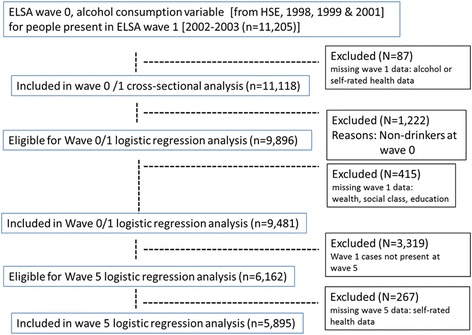
Flowchart illustrating participation in the study

The English Longitudinal Study of Ageing [ELSA] sample is selected to be representative of people aged 50 years and over, living in private households in England [24].The ELSA sample has been shown to be representative of the general population [25]. The ELSA sample was drawn from households that were sampled by the Health Survey for England (HSE) in years 1998, 1999 and 2001 (see Fig. 1). The HSE is an annual cross-sectional household survey and eligible individuals participate in a personal interview followed by a nurse visit [22].

Households were included in ELSA if one or more individual household member was aged 50 or over at the time of the first wave of the ELSA survey, which took place between March 2002 and March 2003. In this analysis, alcohol consumption and frequency data collected in the HSE was used rather than that collected in Wave 1 of ELSA which only collected on information on the frequency of drinking. This time period is referred to as Wave 0 of ELSA. As with a previous study, a combination of Wave 0 and Wave 1 were used to define the baseline period [26]. Wave 5 of ELSA was conducted between June 2010 and June 2011. Ethical approval for all the ELSA waves was granted by the National Research and Ethics Committee. All participants gave informed consent. More information on ELSA can be found at http://www.elsa-project.ac.uk/documentation.

There were 11,205 respondents at Wave 1 who also had related information from Wave 0 (Table 1). 1222 non-drinking respondents were excluded from the logistic regression analyses at wave 0/1. As noted in the introduction, this was because the intention was to evaluate public health guidance for people who drink alcohol and therefore we restricted some of the analysis to people who reported at least some level of alcohol consumption. For the cross-sectional analysis there were 9481 participants. For the longitudinal analysis there were 5895 participants.

**Table 1:** Distribution of drinking profiles at wave 0 and attrition rate at wave 5

	Sample size (Wave 0)	% of participants at wave 0	% Attrition rate at wave 5
Number of participants in Wave 0	11,120	100	
Number of participants in Wave 5	5868		47.2
Non-drinking (never and stopped)	1222	10.9	
Drinking Profile 0-A: “always non-drinker”	602	5.4	60.3
Drinking Profile 0-B: “stopped drinking”	620	5.5	61
Low risk drinking (below threshold: men <21, women <14 units per week)	7684	69	
Drinking Profile 1: Low risk drinking: Occasional-social (monthly or less)	2804	25.2	48.4
Drinking Profile 2: Low risk drinking (less than 4 times per week)	3763	33.8	44.8
Drinking Profile 3: Low risk drinking (almost every day)	1117	10	46
Focal drinking (above threshold but less than 4 times per week)	525	4.7	
Drinking Profile 4: FOCAL DRINKING- above threshold but less than 4 times per week AND hazardous (Men > 21 units; Women > 14 units)	486	4.3	39.9
Drinking Profile 5: FOCAL DRINKING- above threshold but less than 4 times per week AND high risk (Men > 50 units; Women > 35 units	39	0.3	51.3
Heavy drinking (above threshold and almost every day)	1689	15.1	
Drinking Profile 6: HEAVY DRINKING- above threshold and almost every day) AND hazardous (Men > 21 units; Women > 14 units]	1356	12.1	42.3
Drinking Profile 7: HEAVY DRINKING- above threshold and almost every day) AND high risk (Men > 50 units; Women > 35 units)	333	3	49.8

86 participants could not be assigned to a drinking profile at Wave 0 due to missing data

### Variable definition and measurement

#### Exposure to alcohol: drinking profiles

A series of drinking profiles were constructed (see Table 1). The purpose of these profiles was to combine the consumption and the frequency data on drinking. Consumption was measured in relation to three levels as defined by the UK government [21]. Lower-risk drinking was defined as up to 14 units^1^ per week for adult women and 21 units per for adult men. Increasing-risk drinking was defined as being between 15 and 35 units per week and as being between 22 and 50 units per week for adult women and adult men, respectively. Higher-risk drinking was defined as being over 35 units per week (adult women) and over 50 alcohol units per week (adult men).

Based on information about the frequency of drinking in the last 12 months, participants were categorised as: less than monthly, from once a month up to 4 times a week, almost every day or every day. The consumption and frequency data were then combined into seven drinking profiles. Profiles 1–3 are classified as low-risk drinking but with different drinking frequencies. Profiles 4–5 are labelled as “focal drinking” because frequency of drinking is less than four times per week but consumption is classified as increasing-risk drinking. Profiles 6–7 are labelled as “heavy drinking” because frequency of drinking is almost every day and consumption is classified as higher-risk drinking. In addition, two non-drinking profiles (0-A and 0-B) were included in the descriptive statistics but are not included in the logistic models (see Table 1).

#### Covariates

Eight other variables were examined: Wave 0-Gender (male/female), Wave 0-Age (45-64/65-74/75+), Wave 1 - Wealth (5 quintiles from least to most affluent), Wave 1- Social class (manual/intermediate/professional), Wave 1-Education (none/secondary/higher) and Wave 1 - household composition (alone/not alone); Wave 0 Smoking (never smoked/used to smoke occasionally/used to smoke regularly/ current smoker) and Wave 0 Body Mass Index (BMI) (<20, 20–25,26-30, >30).

Wealth quintiles refer to household wealth including financial, physical, and housing wealth, but not pension wealth. Wealth was calculated net of debt and includes the value of any home and other property (less mortgage); financial assets covering all types of savings available in England; the value of any business assets and physical wealth such as artwork and jewellery. Social class was measured using the three class version of the Office of National Statistics (manual/intermediate/professional). For educational qualifications, participants were classifıed into three groups based on the highest qualifıcation achieved: no qualifıcations, indicating that the individual left education without formal qualifıcations; intermediate, which includes participants who have completed high school– equivalent qualifıcations (O-level, A-level, or National Vocational Qualifıcations [NVQs] at levels 1–3); and higher education, including those with college or university degrees or NVQ at Level 4 or 5.

#### Outcome: self-rated health

Participants were asked to rate their health on a five-point scale. This variable was recorded at Wave 0 as 1 = very good, 2 = good, 3 = fair, 4 = bad, 5 = very bad and at wave 5, 1 = excellent; 2 = very good, 3 = good, 4 = fair, 5 = poor. These variables were converted into a two level variable – good/fair and poor – with good/fair health being the sum of responses ranging from “excellent” to “fair”, and poor health the sum of responses ranging “bad”/”poor” to “very bad”. Poor self-rated health has been shown to be a correlate of ill-health and a predictor of mortality among older adults [27, 28]. The validity of self-rated health is indicated by the finding that the correlation between wave 0 self-rated health and wave 0 long standing illness was 0.417 (*p* < .001) and 0.536 (*p* < .001) with limiting long-standing illness. Long standing illness was defined by a positive response to the question, “Do you have any longstanding illness, disability, or infirmity of any kind? By longstanding I mean anything that has troubled you over a period of time or that is likely to affect you over a period of time?” Participants were then asked if long standing illness limited their daily activities such as pushing a vacuum cleaner.

### Analysis

The association between drinking profiles and self-rated health at wave 0 and wave 5 was examined using descriptive statistics and the chi-square test. Logistic regression analyses were undertaken to examine the association between the nine independent variables (drinking profiles and the variables described above) and the dependent variable (self-rated health). The regression analysis was restricted to participants who reported any alcohol consumption in the previous twelve months. All levels of drinking were compared to the lowest level of alcohol consumption (Drinking Profile 1). Unadjusted and adjusted odds ratios and corresponding 95 % CIs were calculated. All *p* values were considered to be statistically significant if less than 0.05. Analyses were undertaken using SPSS version 19.0.

Attrition rates at wave 5 relative to wave 0 were calculated. The attrition rate is the percentage loss of participants between wave 0 and wave 5. The attrition rate was calculated for all the drinking profiles in order to ascertain whether certain drinking profiles were associated with a greater or lesser degree of loss relative to the overall sample, For example, if heavy drinkers had a higher rate of loss, this might indicate that their non-participation at wave 5 was due to ill-health or death.

## Results

Table 1 shows that at Wave 0, 10.9 % of respondents were non-drinkers, of whom half had previously been drinkers (Drinking Profile 0-B), while half had never drunk (Drinking Profile 0-A). The majority of respondents (69 %) reported drinking below the recommended weekly amount. 19.8 % of the sample reported drinking above the recommended level. 5866 (53 %) of Wave 0 respondents were re-interviewed at Wave 5. Table 1 also shows that non-drinkers at Wave 0 had higher attrition rates by Wave 5 (60 % for never drinkers; 61 % for ex-drinkers) compared to the overall sample (47 %) (*X*^2^ = 99.0, *p* < 0.01). Heavy drinkers did not have higher attrition rates compared to the overall sample.

At Wave 0, 9 % of the sample reported poor self-rated health (Table 2). At Wave 0, there was significant variation between the drinking profiles (*X*^2^ = 212.3, *p* < 0.01). Poor self-rated health was highest among those who had stopped drinking, followed by those who never drank. The rates of poor self-rated health among non-drinkers were significantly higher than the rates of poor self-rated health for any of the groups who reported alcohol consumption. At Wave 5, 8.1 % of the sample reported poor self-rated health (Table 2). There was significant variation between the drinking profiles (*X*^2^ = 76.2, *p* < 0.01). At Wave 5, poor self-rated health was highest among those who had never drank followed by those who stopped drinking. For the respondents who had stopped drinking at wave 0 and participated in wave 5, there was a modest reduction in the level of poor self-rated health at wave 5. It should be noted that 61 % of those who had stopped drinking by wave 0 were not present in wave 5. No other drinking profile (among those present at both wave 0 and wave 5) was associated with a reduction in poor self-related health between wave 0 and wave 5. A comparison of stopped drinking vs. always non-drinkers showed that the former were significantly more likely to report an improvement in health status (*X*^2^ = 10.5, p = 0.05). A comparison of stopped drinking vs low risk drinking (drinking profile 3) showed that the former were significantly more likely to report an improvement in health status between waves 0 and 5 (*X*^2^ = 25.2, *p* < 0.01).

**Table 2:** Proportion of wave 0 drinking profiles with poor self-rated health at wave 0

	Wave 0			Wave 5
Wave 0 Drinking Profiles [N and % of total sample]	% of Drinking Profile with Poor Self-rated Health at Wave 0 [N = 11,118]	% of Drinking Profile with Poor Self-rated Health at Wave 0 [N = 5252] *In W0 but not in W5*	% of Drinking Profile with Poor Self-rated Health at Wave 0 [N = 5868] *In W0 and in W5*	% Drinking Profile with Poor Self-rated Health at Wave 5 [N = 5868]
Drinking Profile 0-A	15.5	16.6	13.8	18.3
Drinking Profile 0-B	22.3	26.2	16.2	12.3
Drinking Profile 1	9.8	12.0	7.8	10.8
Drinking Profile 2	7.0	10.1	4.5	6.0
Drinking Profile 3:	7.6	12.3	3.6	6.1
Drinking Profile 4	9.1	10.8	7.0	8.6
Drinking Profile 5	5.1	10.0	0.0	5.3
Drinking Profile 6	5.0	7.7	3.1	5.8
Drinking Profile 7	8.1	8.4	7.8	8.4
ALL valid participants 100 %)	9.0	12.1	6.2	8.1

Further logistic regression analysis of the association between drinking and health was carried out for drinkers only. The results of the unadjusted and adjusted models for Wave 0 are summarised in Table 3, with self-rated health as the dependent variable (the model computed the odds of reporting poor self-rated health against the reference category of fair/good health). For the unadjusted models there were no drinking profiles associated with significantly higher odds of poor self-rated health. In the adjusted models there were again no drinking profiles associated with significantly higher rates of poor self-rated health. However, two profiles (drinking profile 2 and drinking profile 6) were significantly associated with better self-rated health than the reference category in the adjusted models. Those who reported low risk-near daily levels of consumption, focal drinking (increasing-risk or higher-risk) or heavy drinking did not differ significantly from the reference category. The adjusted model suggests that the association between alcohol and self-rated health was not significantly affected by other variables.

**Table 3:** Odds ratios for the association between wave 0 drinking profiles, socio-demographic characteristics and health behaviours and self-rated health at wave 0

Independent variables	Unadjusted				Adjusted^a^			
	Odds ratio	95 % C.I. for OR		Sig. Level	Odds ratio	95 % C.I. for OR		Sig. Level
		Lower	Upper			Lower	Upper	
Reference Category								
Drinking Profile 1								
Drinking Profile 2	0.69	0.58	0.82	<.01	0.83	0.69	1.00	0.05
Drinking Profile 3	0.75	0.59	0.97	0.03	1.07	0.81	1.41	0.64
Drinking Profile 4	0.91	0.65	1.27	0.59	0.81	0.57	1.16	0.26
Drinking Profile 5	0.50	0.12	2.07	0.33	0.34	0.08	1.46	0.15
Drinking Profile 6	0.48	0.37	0.64	<.01	0.66	0.50	0.89	0.01
Drinking Profile 7	0.81	0.53	1.22	0.31	0.71	0.46	1.11	0.13
Wealth. Reference category- wealth quintile 1 [lowest 20 %)								
Wealth quintile 2 (21–40 %)	0.60	0.50	0.73	<.01	0.63	0.52	0.77	<.01
Wealth quintile 3 (41–60 %)	0.29	0.23	0.36	<.01	0.32	0.25	0.40	<.01
Wealth quintile 4 (61–80 %)	0.23	0.18	0.29	<.01	0.27	0.21	0.35	<.01
Wealth quintile 5 (81–100 %-wealthiest)	0.12	0.09	0.16	<.01	0.16	0.11	0.23	<.01
Social class. Reference Category: Manual								
Social class. Intermediate	0.86	0.73	1.03	0.10	1.15	0.95	1.38	0.15
Social class. Professional	0.37	0.30	0.46	<.01	0.85	0.66	1.10	0.22
Education. Reference Category: No qualifications								
Education. Secondary Education	0.54	0.46	0.64	<.01	0.72	0.60	0.86	<.01
Education. Degree or Above	0.27	0.21	0.34	<.01	0.51	0.38	0.68	<.01
Household Size. Reference Category: Living Alone								
Household size: 2 or more people in household	0.68	0.58	0.80	<.01	0.90	0.75	1.08	0.20
Gender. Reference Category: Male								
Gender-Female	0.70	0.60	0.81	<.01	0.58	0.49	0.68	<.01
Age-group. Reference Category: 45–64								
Age-group. 65–74	1.07	0.89	1.27	0.47	0.85	0.70	1.02	0.09
Age-group. 75+	1.25	1.02	1.55	0.03	0.76	0.60	0.97	0.03
Smoking: Reference Category: Never smoked								
used to smoke occasionally	1.26	0.88	1.80	0.20	1.23	0.84	1.81	0.28
used to smoke regularly	1.70	1.41	2.06	<.01	1.41	1.15	1.73	<.01
current smoker	2.62	2.13	3.21	<.01	1.77	1.41	2.20	<.01
BMI: Reference Category: <20								
20–25	0.52	0.32	0.86	0.01	0.80	0.45	1.40	0.43
25–30	0.75	0.47	1.21	0.24	1.09	0.63	1.88	0.76
>30	1.06	0.66	1.72	0.80	1.43	0.82	2.50	0.20

^a^Adjusted for W1-wealth, W1-social class, W1-education, W1-household size, W0-gender, W0-age, W0-smoking, W0-BMI

Wealth had a strong association with self-rated health at Wave 0. This association remained after adjusting for other variables. Women and those with higher education reported better self-rated health. Social class and household size did not have a significant association after adjustment. Smoking and BMI > =25 were associated with poorer self-rated health. Finally, if we turn to consider how drinking profiles were associated with health at Wave 5 (Table 4), the analysis shows that no Wave 0 drinking profile was associated with poor self-rated health at Wave 5 in the adjusted model, though Wave 0 self-rated health and wealth were strongly associated with Wave 5 self-rated health.

**Table 4:** Demographic predictors of wave 5 self-rated health (partially adjusted and fully adjusted models)

Independent variables	Partially-adjusted (For self-rated health)				Fully-adjusted^a^			
	Odds ratio	95 % C.I. for OR		Sig. Level	Odds ratio	95 % C.I. for OR		Sig. Level
		Lower	Upper			Lower	Upper	
Reference category: Drinking Profile 1								
Drinking Profile 2	0.58	0.45	0.75	<.01	0.78	0.59	1.03	0.07
Drinking Profile 3	0.62	0.42	0.92	0.02	0.96	0.64	1.44	0.84
Drinking Profile 4	0.74	0.47	1.19	0.21	1.02	0.62	1.68	0.93
Drinking Profile 5	0.65	0.09	4.92	0.68	0.57	0.07	4.47	0.59
Drinking Profile 6	0.60	0.42	0.86	<.01	0.79	0.54	1.17	0.24
Drinking Profile 7	0.73	0.40	1.33	0.30	0.94	0.50	1.77	0.84
Reference category: Wave 0 self-rated health (bad/very bad)								
Wave 0 self-rated heath (fair to very good)	10.0	7.69	13.13	<.01	7.29	5.45	7.98	<.01
Reference category: Wealth quintile 1 (lowest 20 %)								
Wealth quintile 2 (21–40 %)	0.51	0.38	0.67	<.01	0.74	0.54	1.01	0.67
Wealth quintile 3 (41–60 %)	0.29	0.22	0.41	<.01	0.56	0.39	0.76	<.01
Wealth quintile 4 (61–80 %)	0.22	0.16	0.31	<.01	0.51	0.35	0.75	<.01
Wealth quintile 5 (81–100 %-wealthiest 20 %)	0.17	0.12	0.24	<.01	0.48	0.32	0.73	<.01

^a^Adjusted for W1-wealth (shown in table), W1-social class, W1-education, W1-household size, W0-gender, W0-age, W0-smoking, W0-BMI

## Discussion

As in previous studies, the prevalence of poor self-rated health was highest among non-drinkers, particularly those who had stopped drinking [16, 29]. Cross-sectional analyses suggest that none of the drinking profiles examined in this study were associated with poor self-rated health and drinking profiles did not predict self-rated health at ten year follow up. Older adults who stopped drinking prior to the study were the only drinking profile associated with an improvement in self-rated health over the ten year period. Demographic variables were independently associated with self-rated health but did not substantially modify the association between alcohol and self-rated health.

A previous study reported similar findings, when the reference category was non-drinkers [27]. This study extends the finding to the case where the reference category was occasional drinkers. However the validity of results obtained from observational studies, such as the one reported here have been questioned due to residual confounding or selection bias [30]. One particular source of bias is wealth [31]. This study confirms that wealth have a strong association with alcohol consumption and we took account of this variable when analysing the association between alcohol consumption and self-rated health. With regards to selection bias we were able to draw on both cross-sectional and longitudinal data. This enabled us to study participants in terms of their baseline drinking profiles and follow those who participated in subsequent waves over a ten year period. Furthermore, the analysis of attrition rates suggests that the longitudinal data was not affected by higher drop-out rates among those with higher levels of baseline drinking. Thus the current analyses go some way towards alleviating concerns on these two issues.

Another finding was that those who stopped drinking by wave 0 but participated in wave 5 were more likely to report improved health ten years later than those who never drank or drank occasionally. This suggests that health may improve as a result of stopping drinking. However we were not able to explore this further as our analysis considers drinking in later life and does not take into account earlier life experiences. Thus to fully understand the association between drinking and health it is necessary to consider the development of alcohol profiles over the life course [32].

Several limitations of the current study should be noted. First, some of the socio-demographic information was collected at a later date (wave 1) than the alcohol consumption data (wave 0). However, this is unlikely to substantially affect the results because these variables represent factors that are stable over time, i.e. wealth, social class, education. The one exception might be household composition. However household composition was not a significant predictor of self-rated health in the adjusted logistic regression model. Second, residual confounding is a common problem in cohort studies due to unmeasured confounders and misclassification. While we have controlled for a wide range of variables, other factors such as physical activity, diet or total energy intake could interact with alcohol consumption and this is potentially an issue that requires further investigation. Third, while there are well-known issues regarding self-reported alcohol consumption, this measure has acceptably validity for the purposes of categorising people into distinct consumption groups [33].

## Conclusions

The results of this study show that in older people who have not stopped drinking, level of alcohol consumption is not associated with self-rated health. Where does this leave clinical advice that responsible weekly drinking levels should be lower in later life [3]? One rationale of drinking guidelines is that responsible drinking is protective against alcohol problems, and this has been demonstrated at older ages [34]. An American study of drinking behaviour after the age of 50 [32] noted that little is known about the longitudinal development of problem drinking but that problem drinking among older adults is usually diagnosed in early adulthood [35]. Together with the present findings, this has implications for public health advice for those aged 50 and over. In particular it suggests that age-specific guidelines might be less appropriate than advice which emphasises a life course approach taking into account health status. It must also be born in mind that few older people consume very large amounts of alcohol. At a population level, alcohol consumption also needs to be considered within the context of existing health and wealth which are the strongest longitudinal predictors of self-rated health.

## Footnotes

1 In the UK one alcohol unit is measured as 10 ml or 8 g of pure alcohol. This equals one 25 ml measure of whisky (ABV 40 per cent); 1/3 of a pint of beer (ABV ≈ 5 per cent) or half a standard (175 ml) glass of red wine (ABV 12 per cent).
